# Inverse Correlation between *pks*-Carrying *Escherichia coli* Abundance in Colorectal Cancer Liver Metastases and the Number of Organs Involved in Recurrence

**DOI:** 10.3390/cancers16173003

**Published:** 2024-08-29

**Authors:** Yasuyuki Shigematsu, Rumiko Saito, Hiroaki Kanda, Yu Takahashi, Kengo Takeuchi, Shunji Takahashi, Kentaro Inamura

**Affiliations:** 1 Department of Pathology, Cancer Institute Hospital, Japanese Foundation for Cancer Research (JFCR), 3-8-31 Ariake, Koto-ku, Tokyo 135-8550, Japan; kentakeuchi-tky@umin.net; 2 Division of Pathology, Cancer Institute, JFCR, 3-8-31 Ariake, Koto-ku, Tokyo 135-8550, Japan; 3 Department of Medical Oncology, Cancer Institute Hospital, JFCR, 3-8-31 Ariake, Koto-ku, Tokyo 135-8550, Japan; rumiko.saito@jfcr.or.jp (R.S.); s.takahashi-chemotherapy@jfcr.or.jp (S.T.); 4 Department of Clinical Chemotherapy, Cancer Chemotherapy Center, JFCR, 3-8-31 Ariake, Koto-ku, Tokyo 135-8550, Japan; 5 Graduate School of Engineering, Chiba Institute of Technology, 2-17-1 Tsudanuma, Narashino, Chiba 275-0016, Japan; 6 Department of Pathology, Saitama Cancer Center, 780 Komuro, Ina, Kita-adachi-gun, Saitama 362-0806, Japan; hkanda@saitama-pho.jp; 7 Division of Hepatobiliary and Pancreatic Surgery, Cancer Institute Hospital, JFCR, 3-8-31 Ariake, Koto-ku, Tokyo 135-8550, Japan; yu.takahashi@jfcr.or.jp; 8 Pathology Project for Molecular Targets, Cancer Institute, JFCR, 3-8-31 Ariake, Koto-ku, Tokyo 135-8550, Japan; 9 Division of Tumor Pathology, Jichi Medical University, 3311-1 Yakushiji, Shimotsuke, Tochigi 329-0431, Japan

**Keywords:** colorectal cancer, polyketide synthetase gene (*pks*), *Escherichia coli*, liver metastasis, systemic inflammation, tumor-immune microenvironment

## Abstract

**Simple Summary:**

This study explores whether *Escherichia coli* carrying the polyketide synthetase (*pks*) gene cluster in colorectal cancer (CRC) liver metastasis tissues influence immune responses and cancer recurrence patterns. Analyzing tissues from 239 patients, we found that *pks^+^ E. coli* was present in 66.7% of liver metastasis cases. Higher levels of *pks^+^ E. coli* were associated with lower levels of the serum C-reactive protein, fewer densities of CD4^+^ cells and CD163^+^ cells in the tumor microenvironment, and a reduction in the number of organs affected by recurrent metastasis. These findings suggest that *pks^+^ E. coli* may influence both systemic and local immune responses, potentially reducing the diversity of affected metastatic organs.

**Abstract:**

Colibactin, a genotoxin produced by *Escherichia coli* strains harboring the polyketide synthetase (*pks*) gene cluster, causes DNA damage and somatic mutations. *pks*^+^ *E. coli* is enriched in primary colorectal cancer (CRC) and is associated with clonal driver mutations, but its role in CRC liver metastasis is unclear. We assessed the association of *pks^+^ E. coli* in CRC liver metastasis tissues with systemic and local immune responses and the number of organs involved in recurrence using specimens and clinicopathological data from 239 patients with CRC liver metastasis who underwent metastasectomy. The levels of *pks*^+^ *E. coli* in fresh-frozen specimens were quantified as “very low” (<50th percentile), “low” (50th to 75th percentiles), and “high” (>75th percentile) using a digital PCR. Immunohistochemical analysis of tumor-infiltrating immune cells was performed using tissue microarrays. Systemic inflammation was evaluated using serum C-reactive protein (CRP) levels. *pks*^+^ *E. coli* was detected in 66.7% (157 of 239) liver metastasis tissues. Higher levels of *pks*^+^ *E. coli* were associated with decreased serum CRP levels and reduced densities of CD4^+^ cells and CD163^+^ cells in the tumor-immune microenvironment. The “high” *pks^+^ E. coli* group had fewer metastatic organs involved than the “very low” *pks^+^ E. coli* group (mean number of organs: 1.00 vs. 1.23). These findings suggest that *pks*^+^ *E. coli* play a modulating role in CRC metastasis.

## 1. Introduction

Colorectal cancer (CRC) is one of the most prevalent cancers worldwide and is a leading cause of cancer-associated mortality. Liver metastasis develops in approximately 20–30% of patients during the course of CRC [1], and has a 5-year survival rate of less than 5% in cases in which curative surgery is not possible [2,3,4]. Even in patients undergoing curative-intent resection for liver metastases, recurrence is a major concern, occurring in 80–85% of cases [5,6,7]. Therefore, the factors contributing to the metastatic progression of CRC, especially factors within the hepatic microenvironment, need to be clarified.

*Escherichia coli* strains of the B2 phylotype commonly harbor a pathogenicity gene cluster termed polyketide synthases (*pks*) that encodes enzymes for colibactin biosynthesis [8,9,10]. Experimental studies have shown that colibactin can alkylate DNA, induce DNA double-strand breaks, and cause specific somatic mutations in human cells [11,12,13]. Previous studies found that the primary CRC increased numbers of adherent *E. coli* compared with non-neoplastic colonic mucosa, and the presence of *pks*^+^ *E. coli* is associated with an increase in clonal driver mutations and lower disease stages [14,15]. In addition, evidence suggests that *pks*^+^ *E. coli* suppresses the host immune response in the tumor-immune microenvironment (TIME) of the primary site [16]. However, the role of *pks*^+^ *E. coli* in the TIME of liver metastasis tissues and its effect on recurrence patterns are unclear.

The hepatic TIME provides premetastatic niches that support CRC cell implantation and proliferation [17,18]. Concurrently, systemic inflammation promotes CRC progression and metastasis by inducing angiogenesis and increasing vascular permeability [19]. Elevated serum C-reactive protein (CRP) levels have been identified as an adverse prognostic indicator in CRC liver metastasis [20]. Additionally, the prognosis in metastatic CRC is associated with the number of organs with metastases [21]. Despite these insights into the complex interplay between the hepatic TIME, systemic inflammation, and CRC liver metastasis, the underlying mechanisms remain unclear and require investigation.

This study evaluated the association of *pks*^+^ *E. coli* in CRC liver metastasis tissues with systemic and local immune responses and the diversity of metastatic organs involved in recurrence. Using fresh-frozen samples to quantify *pks* DNA levels, formalin-fixed paraffin-embedded (FFPE) specimens for evaluating the TIME and clinical data of serum CRP levels and metastatic organs involved in recurrence after R0 metastasectomy were used to explore the potential role of *pks*^+^ *E. coli* in the complex dynamics of CRC liver metastasis.

## 2. Materials and Methods

### 2.1. Patients and Specimens

Specimens from surgical resection were collected from a series of patients diagnosed with CRC who underwent R0 liver metastasectomy at the Cancer Institute Hospital in Tokyo, Japan, between January 2005 and December 2015. These specimens, sourced from metastatic liver sites, were then segregated and conserved as fresh-frozen samples and in FFPE blocks. The inclusion criteria included having ample fresh-frozen and FFPE samples available in addition to comprehensive pathological reports. Detailed clinicopathological information, including age, sex, preoperative blood tests, tumor site, the number of liver metastases, chemotherapy history, and the number of organs involved at the time of relapse, was collated from digital medical records. The organs evaluated as sites of metastasis and recurrence include the brain, lungs, liver, adrenal glands, peritoneum, spleen, bones, and lymph nodes. The medical history of each patient was carefully reviewed by the research team. Ethical approval was granted by the Ethics Committee of the Japanese Foundation for Cancer Research (approval number: 2016-1087), and the requirement for informed consent was waived owing to the retrospective nature of the investigation.

### 2.2. Pathological Analysis

Sections of tissue, 4 μm thick each, derived from FFPE samples, were subjected to staining with hematoxylin and eosin. The diagnosis of CRC liver metastasis was validated by two pathologists (Y.S. and K.I.) based on the criteria of the 5th edition of the World Health Organization guidelines [22].

#### 2.2.1. Tissue Microarray

To immunohistochemically assess the tumor and associated tumor-infiltrating cells, a tissue microarray was constructed using FFPE tumor samples as previously described [23]. Specifically, the targeted areas were cored from the donor paraffin blocks using a 2 mm needle, and these samples were subsequently arranged into a recipient block using a KIN-1 manual tissue arrayer (Azumaya, Tokyo, Japan). For each tumor, three core samples that best represented the predominant histological features were selected. The tumor microarray sections, each 4 µm thick, were prepared for immunohistochemical analysis, as described below.

#### 2.2.2. Immunohistochemistry

Immune cell infiltrations in the TIME were evaluated using anti-CD8 (1:3; clone C8/144B; catalog number 413201; Nichirei, Tokyo, Japan), anti-CD4 (1:2; clone 4B12; catalog number 413951; Nichirei), anti-CD20 (1:800; clone L26; catalog number NCL-L-CD20-L26; Leica Biosystems, Newcastle, UK), anti-FOXP3 (1:100; clone 236A/E7; catalog number ab20034; Abcam, MA, USA), anti-CD163 (1:1200; clone 10D6; catalog number CD163-L-CE; Leica Biosystems), and anti-CD68 (1:1000, clone KP-1; DAKO, catalog number M0814; Glostrup, Denmark) mouse monoclonal antibodies. For the CD68 antigens, a 5 min incubation with a 200-fold dilution of protease K was performed. Other antigens were activated by heating at 100 °C for 20 min in an ethylenediaminetetraacetic acid (EDTA)-surfactant buffer (pH 9.0). Non-cancerous liver tissue in the same tissue microarray section, including hepatocytes, bile ducts, lymphocytes, and nerves, was used as positive and negative controls. Immunostaining was performed using a Bond-III automated staining system (Leica Microsystems, Buffalo Grove, IL, USA), and antigens were detected using a Bond Polymer Refine Detection Kit (Leica Microsystems).

#### 2.2.3. Evaluation of Immunohistochemistry (IHC) Variables in Tissue Microarrays

To quantify the number of immune cells infiltrating tumors, immunostained sections were digitized and analyzed as previously described [24,25]. Briefly, immunostained sections at ×40 magnification were scanned with a NanoZoomer Digital Pathology System (Hamamatsu Photonics KK, Shizuoka, Japan) at a resolution of 0.55 pixel/μm. The images were semiautomatically digitized using the proprietary NanoZoomer Digital Pathology Image file format. Fiji, an open-source platform for biological-image analysis [26], was used to quantify the number of immune cells within the tumors. A representative microscopic field from each tissue core was chosen from the scanned tumor microarray images, and Fiji was used for the analysis. Three tissue cores were analyzed per tumor, and the mean number of immune cells was calculated to represent the immune cell infiltration in the tumor.

### 2.3. DNA Extraction and Digital PCR to Quantify Polyketide Synthase (pks)

Genomic DNA was isolated from fresh-frozen CRC liver metastasis tissue specimens using the NucleoSpin Tissue Kit (Takara Bio Inc., Shiga, Japan) and quantified using a Nanodrop ND-1000 spectrophotometer (Thermo Fisher Scientific Inc., Wilmington, DE, USA). The initial polymerase chain reaction (PCR) was conducted using 25 μL of the sample solution comprising 100 ng of extracted DNA and specific primers designed to detect *polyketide synthase (pks)*-specific sequences (Table S1). The initial PCR assay for *pks* involved an initial denaturation step at 95 °C for 10 min, followed by 40 cycles with denaturation at 95 °C for 15 s, followed by annealing at 57 °C for 30 s, and extension at 72 °C for 30 s. For quantification, the initial PCR product of *pks* was diluted 10^6^-fold and subjected to an EvaGreen-based Droplet Digital PCR using 1 μL of the diluted sample. To determine the relative amount of *pks* DNA in a sample based on the quantity of gDNA input, an EvaGreen-based Droplet Digital PCR was performed on samples comprising 10 ng of gDNA with specific primers for the reference human gene *SLCO2A1* (Table S1). The EvaGreen-based Droplet Digital PCR conditions for *pks* included an initial denaturation step at 95 °C for 10 min, followed by 40 cycles with denaturation at 95 °C for 15 s, followed by annealing at 57 °C for 30 s, and extension at 72 °C for 30 s, and *SLCO2A1* included an initial denaturation step at 95 °C for 10 min, followed by 35 cycles with denaturation at 95 °C for 15 s, followed by annealing and extension at 60 °C for 1 min. The Droplet Digital PCR was performed using the QX200 Droplet Digital PCR System (Bio-Rad Laboratories, Inc., Hercules, CA, USA) and EvaGreen assays. To ensure the accuracy and reliability of *pks* DNA quantification, we adhered to the recommended contamination control guidelines for low-biomass microbiome research [27].

### 2.4. Statistical Analysis

Statistical analyses were conducted using R software version 4.2.3 (R Foundation for Statistical Computing, Vienna, Austria). For analysis, cases were categorized into high, low, and very low groups according to the *pks* DNA levels. Welch’s *t*-test was used to assess the differences in mean values between the two groups, whereas one-way analysis of variance (ANOVA) was used for comparisons among the three groups. Differences in the median values among the three groups were evaluated using the Kruskal–Wallis test. To account for multiple comparisons and reduce the risk of a type I error, Bonferroni correction was applied to the *P* values obtained from the pairwise comparisons. The chi-squared test was employed to evaluate the relationship between the categorized number of metastatic sites and the different *pks^+^ E. coli* groups. Ordinal logistic regression was used to examine the relationship between *pks^+^ E. coli* DNA quantities in liver metastases (as an ordinal independent variable) and the density of tumor-infiltrating immune cells, with statistical significance set as two-sided *p* values less than 0.05.

## 3. Results

### 3.1. Detection of pks^+^ E. coli in CRC Liver Metastasis Tissues

We examined liver metastasis tissues from 239 patients with CRC and detected *pks^+^ E. coli* DNA in 159 cases (66.7%). To assess the relationship between *pks^+^ E. coli* abundance and clinicopathological features, we stratified the cases into three groups based on the amount of *pks^+^ E. coli* DNA present in the liver metastasis tissue: “very low” (below the 50th percentile; N = 201), “low” (between the 50th and 75th percentiles; N = 19), and “high” (above the 75th percentile; N = 19). Patients’ age, sex, and primary tumor location did not differ significantly according to the amount of *pks^+^ E. coli* DNA present (Table S2).

### 3.2. Systemic Immune Responses

**Figure 1:**
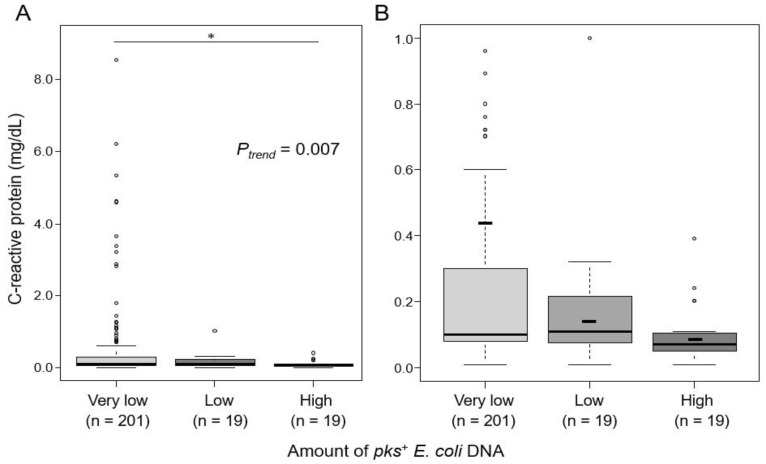
Box plots illustrating the trend in C-reactive protein (CRP) levels grouped by the amount of *pks*^+^ *E. coli* DNA present. (**A**) The box plots indicate a trend in preoperative serum CRP levels across the different *pks*^+^ *E. coli* DNA groups (*P*_trend_ = 0.007), suggesting a negative association between the amount of *pks*^+^ *E. coli* DNA in colorectal cancer liver metastasis tissues and systemic inflammation. (**B**) An enlarged view of the CRP range 0–1.0 mg/dL highlights the differences between the *pks^+^ E. coli* DNA groups. The short horizontal line within each box plot represents the mean value. * indicates *p* < 0.05.

The level of CRP, an indicator of systemic inflammation, was inversely associated with the amount of *pks^+^ E. coli* DNA in CRC liver metastasis tissues (*P*_trend_ = 0.007; Figure 1). The “high” *pks^+^ E. coli* group showed lower CRP levels than the “very low” *pks^+^ E. coli* group (median: 0.07 vs. 0.10 mg/dL; *p* = 0.013). Within the “Very low” group, no comorbidities known to substantially influence CRP levels were observed, aside from the patients’ primary diseases. In a secondary analysis, we separated the “Very low” group into “None” (N = 80) and “Detectable low” (N = 121) groups. The analysis across the four groups—”None”, “Detectable low”, “Low”, and “High”—revealed similar trends (Figure S1). These data indicate an inverse association between the presence of *pks^+^ E. coli* at metastatic sites and systemic inflammation.

### 3.3. Tumor-Immune Microenvironment (TIME)

**Figure 2:**
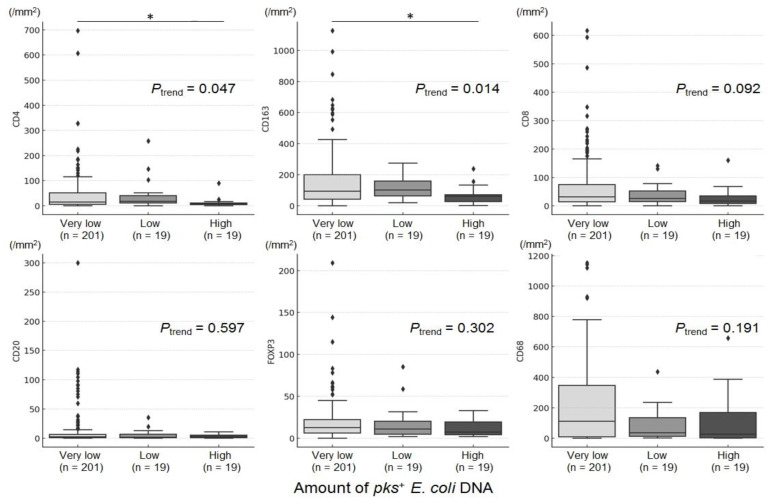
Box plots illustrating the density of various immune cell markers stratified by the amount of *pks^+^ E. coli* DNA. The boxplots depict the distribution of six different immune cell markers (CD4, CD163, CD8, CD20, FOXP3, and CD68) across three distinct groups of *pks^+^ E. coli* DNA (“very low”, “low”, and “high”). The horizontal axis represents the groups categorized by the amount of *pks^+^ E. coli* DNA in colorectal cancer liver metastasis tissues, whereas the vertical axis represents the density of tumor-infiltrating immune cells. * indicates *p* < 0.05.

Within the tissue microarray tissue cores, tumor-infiltrating immune cells showed relatively consistent patterns and were characterized as either dispersed or clustered. Immunohistochemical characterization identified these cells as CD8^+^, CD4^+^, CD20^+^, FOXP3^+^, CD163^+^, or CD68^+^ cells, and the density of each cell type in the tumor-immune microenvironment (TIME) was quantified (Figure S2). The densities of CD4^+^ and CD163^+^ cells decreased with an increase in *pks*^+^ *E. coli* DNA in CRC liver metastasis tissues (*P*_trend_ = 0.047 for CD4^+^ cells and *P*_trend_ = 0.014 for CD163^+^ cells; Figure 2). Compared with the “very low” *pks^+^ E. coli* group, the “high” *pks^+^ E. coli* group had fewer CD4^+^ cells (*p* = 0.033) and CD163^+^ cells (*p* = 0.023) in the TIME. Conversely, no significant trends or differences were observed in the densities of other immune cell types, such as CD8^+^, CD20^+^, FOXP3^+^, or CD68^+^ cells, between groups stratified by *pks*^+^ *E. coli* DNA levels. The secondary analysis, with “Very low” split into “None” and “Detectable low”, revealed similar trends across the four groups in tumor-infiltrating immune cells, with the CD163 category maintaining statistical significance (Figure S3).

### 3.4. Diversity of Metastatic Organs Involved in Recurrence

To assess the likelihood of recurrent extracolonic metastasis following R0 resection with hepatic metastasectomy in patients who experienced recurrence, we evaluated the number of organs involved in 152 such cases. The distribution of cases among the “very low”, “low”, and “high” *pks^+^ E. coli* groups was 128, 13, and 11, respectively. A significant inverse association was observed between the amount of *pks^+^ E. coli* present in liver metastasis tissue and the number of metastatic organs involved in recurrence (*p* = 0.034; Table 1). Specifically, the “high” *pks^+^ E. coli* group had a significantly lower number of metastatic organs involved than the “very low” *pks^+^ E. coli* group, with metastasis confined to the liver in all 11 cases of recurrent metastasis following R0 resection with hepatic metastasectomy in the “high” *pks^+^ E. coli* group (mean number of organs: 1.00 vs. 1.23 in the “high” vs. “very low” *pks^+^ E. coli* groups; Welch’s *t*-test, *p* < 0.00001; and Bonferroni-adjusted *p* < 0.00001). A secondary analysis that subdivided the “Very low” group into “None” and “Detectable low” categories showed similar trends (Table S3).

## 4. Discussion

This study provides new insights into the complex associations between *pks*^+^ *E. coli* DNA levels in CRC liver metastasis tissues and systemic immune responses, the TIME, and patterns of metastatic recurrence. Using a highly sensitive method to detect *pks*^+^ *E. coli* DNA, we confirmed its presence in liver metastatic sites. By categorizing the samples into three groups based on the amount of *pks*^+^ *E. coli* DNA present in metastasis tissue, we found associations with both systemic and local immune responses. Specifically, higher amounts of *pks*^+^ *E. coli* DNA were associated with reduced systemic inflammation, as indicated by lower serum CRP levels and alterations in the TIME and by the reduced numbers of CD4^+^ and CD163^+^ cells. Additionally, the number of metastatic organs involved in recurrence was inversely associated with the amount of *pks*^+^ *E. coli* DNA present. These associations suggest that *pks*^+^ *E. coli* may modulate immune responses, thereby contributing to a decreased diversity of metastatic organs in CRC metastatic tumors harboring *pks*^+^ *E. coli*. Our findings provide a new perspective on the interplay between *pks*^+^ *E. coli* and host immunity in the complex dynamics of CRC metastasis.

The intratumoral microbiota can influence metastatic tropism partly by modulating the TIME [28,29,30]. Specifically, by inducing innate immune cells, including macrophages, intratumoral microbiota contributes to the formation of a premetastatic niche, a favorable microenvironment for metastatic cell implantation and proliferation [28]. In this study, the amount of *pks*^+^ *E. coli* DNA present in CRC liver metastases was inversely associated with the number of organs affected by recurrent metastases. This finding suggests that a high load of *pks*^+^ *E. coli* may influence metastatic behavior, potentially limiting the spread to fewer organs. Additionally, a high abundance of *pks*^+^ *E. coli* was associated with reduced densities of CD4^+^ T cells and CD163^+^ M2-type macrophages in metastatic tissues. Interestingly, similar patterns were noted for other lymph cell types, including CD8^+^ cells, implying that a reduction in CD4^+^ cells may indirectly contribute to this effect. CD163^+^ M2-type macrophages promote tumor growth, invasion, and metastasis. Furthermore, a recent study using mouse models found that intratumoral *E. coli* reduces the density of M2-type macrophages in the TIME via the Toll-like receptor 4 pathway [31]. Emerging evidence has also shown that CD163^+^ M2-type macrophages play a role in creating an immunosuppressive, pro-angiogenic, and pro-invasive TIME [32,33,34]. The observed decrease in these cell types may indicate a shift toward an immunocompetent TIME, potentially hindering metastatic spread. The potential effect of TIME modulation by *pks^+^ E. coli* on metastatic trophism warrants further investigation.

The evaluation of CD4^+^ T cells, CD8^+^ T cells, FOXP3^+^ regulatory T cells, CD20^+^ B cells, CD68^+^ macrophages, and CD163^+^ macrophages provides valuable insights into the TIME that may influence patient outcomes [35,36,37,38]. The integration of these markers, like in studies with immunoscore-metastatic and immunoscore-macrophage models, has consistently shown a better prognostic stratification, emphasizing their collective influence compared to individual markers alone [37]. While the exact mechanisms by which *pks^+^ E. coli* modulates the TIME remain unclear, this represents a promising avenue for future research, potentially uncovering novel therapeutic targets and strategies to enhance anti-tumor immunity.

Intratumoral microbiota modulates systemic immune responses [29,39,40], which may influence metastatic trophism. For example, a study using CRC mouse models showed that intratumoral *Fusobacterium nucleatum* upregulates several inflammatory cytokines in serum [39]. In this study, we found an inverse association between the abundance of *pks^+^ E coli* and serum CRP levels. This finding suggests that *pks*^+^ *E. coli* may suppress tumor-induced tissue destruction and progression, potentially limiting metastatic spread to fewer organs. Further studies are warranted to investigate whether and how *pks*^+^ *E. coli* modulates immune responses and influences metastatic organ tropism. Insights from such studies could help develop potential strategies that target *pks*^+^ *E. coli* and related immune pathways, thereby advancing the management of metastatic CRC.

This study has several limitations. First, this is a retrospective analysis and, thus, cannot establish causality. Second, *pks*^+^ *E. coli* DNA was quantified using fresh-frozen specimens obtained from one liver metastasis per patient. Although this method might not reflect the full variability across all metastatic lesions, efforts were made to reduce sampling bias by using tissues from the largest metastatic lesion for analysis. Third, the use of tissue microarrays for immunohistochemical analysis has inherent limitations owing to intra-tissue heterogeneity, which may not accurately represent the entire tumor. To mitigate this, we examined three tissue cores per tumor to capture a wider spectrum of the TIME. Fourth, the mechanisms underlying the observed associations remain unclear and require further investigation. Fifth, although we acknowledge the limitation of not directly assessing *pks* gene expression, the significant findings observed, despite the potential for underestimation due to non-expressing *pks* gene, highlight the importance of the association between *pks^+^ E. coli* and clinicopathological features of CRC liver metastasis. Future investigations incorporating expression analysis could further refine our understanding of the role of *pks^+^ E. coli* in CRC liver metastasis. Sixth, our study primarily focused on characterizing the T cell, B cell, and M2-type macrophage populations within the TIME. Future studies incorporating other immune cell types, including neutrophils and M1-type macrophages, can provide a more comprehensive understanding of the association between *pks^+^ E. coli* and the immune landscape in CRC liver metastasis. Seventh, the observed heterogeneity in immune cell populations within the “Very low” *pks^+^ E. coli* group warrants further investigation to fully elucidate the complex interplay between the microbiome, tumor cells, and host immune responses that shape the TIME. Finally, because our cohort was derived from a single cancer hospital in Japan, the generalizability of our findings to other populations is uncertain. Therefore, larger prospective studies are required to validate our findings.

## 5. Conclusions

This study identified an inverse association between the abundance of *pks*^+^ *E. coli* in CRC liver metastases and the number of metastatic organs involved in recurrence. These findings suggest that this association may be influenced by the modulation of the TIME in metastatic sites and systemic immune responses. Further research is required to understand the clinical implications of these findings and the potential for developing targeted treatment strategies for the management of metastatic CRC.

## Figures and Tables

**Table 1:** Number of organs with metastases according to the level of *pks^+^ E. coli* present in colorectal cancer liver metastasis tissues.

Number of Organs Involved	Amount of *pks^+^ E. coli* DNA			*p*
	Very Low (N = 128)	Low (N = 13)	High (N = 11)	
1	105 (82.0)	10 (76.9)	11 (100)	0.034
2	17 (13.3)	1 (7.7)	0 (0)	
3	6 (4.7)	1 (7.7)	0 (0)	
4	0 (0)	0 (0)	0 (0)	
5	0 (0)	1 (7.7)	0 (0)	

Data presented as N (%).

## Data Availability

The datasets used and/or analyzed during the current study are available from the corresponding author on reasonable request.

## Supplementary Materials

The following supporting information can be downloaded at https://www.mdpi.com/article/10.3390/cancers16173003/s1. Table S1: Primer sequences of *pks* (polyketide synthases) and *SLCO2A1*; Table S2: Clinical features based on the amount of *pks*^+^ *E. coli* in patients with colorectal cancer liver metastasis; Table S3. Recurrent organ diversity associated with *pks*^+^ *E. coli* in colorectal cancer liver metastasis: A four-group analysis; Figure S1: Box plots demonstrating the trend in C-reactive protein (CRP) levels stratified by *pks^+^ E. coli* DNA abundance, with the “Very low” group further subdivided into “None” and “Detectable low”; Figure S2: Representative histologic images of immune cell markers. Immunohistochemical staining of tumor-infiltrating immune cells for CD4, CD163, CD8, CD20, FOXP3, and CD68; Figure S3: Box plots demonstrating the distribution of immune cell markers stratified by *pks^+^ E. coli* DNA levels, further subdividing the “Very low” group into “None” and “Detectable low”.
