# Supplementary material for: Inverse Correlation between pks-Carrying Escherichia coli Abundance in Colorectal Cancer Liver Metastases and the Number of Organs Involved in Recurrence
Source: Cancers (Basel). 2024 Aug 29;16(17):3003. doi: 10.3390/cancers16173003 (PMC11394077; doi:10.3390/cancers16173003)
Supplement: Supplementary file 1 [file cancers-16-03003-s001.zip › cancers-3161887-supplementary.pdf]

Inverse correlation between *pks*-carrying *Escherichia coli* abundance in colorectal cancer liver metastases and the number of organs involved in recurrence

Supplementary tables

Table S1. Primer sequences of *pks (polyketide synthases)* and *SLCO2A1*.

Gene name	Forward primer F (5' –3')	Reverse primer R (5' –3')
Pks	GCAACATACTCGCCCAGACT	TCTCAAGGCGTTGTTGTTTG
SLCO2A1	ATCCCCAAAGCACCTGGTTT	AGAGGCCAAGATAGTCCTGGTAA

Table S2. Clinical features based on the amount of *pks+ E. coli* in patients with colorectal cancer liver metastasis.

**Variable**	**Total (N = 239)**	**Amount of *pks*⁺ *E. coli* DNA**			***P***
		**Very low (N = 201)**	**Low (N = 19)**	**High (N = 19)**	
Age (years) †	62.4 (± 11.1)	62.9 (± 11.1)	57.5 (± 8.0)	62.4 (± 13.0)	0.13
Sex §					0.14
Female	87 (36.4)	76 (37.8)	3 (15.8)	8 (42.1)	
Male	152 (63.6)	125 (62.2)	16 (84.2)	11 (57.9)	
Primary tumor location §					0.41
Right side	50 (20.9)	45 (22.4)	2 (10.5)	3 (15.8)	
Left side	189 (79.1)	156 (77.6)	17 (89.5)	16 (84.2)	
Tumor differentiation §					0.88
Well to moderate	227 (95.4)	190 (95.0)	18 (94.7)	19 (100)	
Poor	11 (4.6)	10 (5.0)	1 (5.3)	0 (0)	
Timing of metastasis §					0.62
Synchronous	132 (55.2)	111 (± 55.2)	12 (± 63.2)	9 (± 47.7)	
Metachronous	107 (44.8)	90 (± 44.8)	7 (± 36.8)	10 (± 52.6)	
History of chemotherapy §					0.49
Absent	79 (33.1)	68 (33.8)	7 (36.8)	4 (21.1)	
Present	160 (66.9)	133 (66.2)	12 (63.2)	15 (78.9)	

§ Data presented as N (%); † Data presented as mean (± standard deviation).
